# Study on Pressure-Bearing Patterns of Gel Plugging Agents in Multi-Scale Fractures

**DOI:** 10.3390/gels11040305

**Published:** 2025-04-21

**Authors:** Shuanghu Si, Yifei Liu, Yinghui Jiang, Chenwei Zou, Ning Yang, Dongfang Lv, Xizhuo Miao, Caili Dai

**Affiliations:** 1 State Key Laboratory of Deep Oil and Gas, China University of Petroleum (East China), Qingdao 266580, China; sishuanghu@oulook.com (S.S.); yangning0332@163.com (N.Y.); lvdongfang20201@163.com (D.L.); miaoxizhuo@outlook.com (X.M.); 2 School of Petroleum Engineering, China University of Petroleum (East China), Qingdao 266580, China; 3 China Petroleum and Natural Gas Co., Ltd. Yumen Oilfield Huanqing Branch, Qingyang 745700, China; ymjiangyh@petrochina.com.cn; 4 Tianjin Branch of CNOOC (China) Co., Ltd., Tianjin 300450, China; zou_chenwei@outlook.com

**Keywords:** fractured reservoir, TRG, pressure-bearing, influence chart, application experiment

## Abstract

In fractured reservoirs, fractures serve as both water channeling and oil flow channels. Because of the impact of bottom water coning, the water channeling phenomenon becomes more problematic in the middle and late stages of reservoir development. Furthermore, residual oil is limited to small-scale fractures. In multi-scale fractures, the conventional pressure-bearing pattern of plugging agents is ambiguous. This results in low oil recovery, low sweep efficiency from water flooding, and low plugging agent application efficiency. Until now, the pressure-bearing patterns related to gel strength in multi-scale fractures have not been clear. In this paper, the gelation performances of temperature-resistant gel (TRG) samples with different elastic moduli were investigated. The elastic modulus of the TRG was normalized by the elastic modulus (G′) and viscosity modulus (G″). Subsequently, we carried out research on the bottom water pressure patterns of TRGs. This study revealed the pressure-bearing patterns of the TRGs under multi-scale fractures. A corresponding influence pattern chart was established, and the optimal surface function was fitted using the MATLAB nonlinear surface data fitting method. Finally, an application experiment for the characteristic chart was carried out. The plugging rate was evaluated based on the permeability reduction and pressure differential across the core samples before and after gel injection. Subsequently, a TRG with certain elastic moduli before and after plugging the core fracture node was selected from the chart. The elastic modulus of the TRG at the injection node prior to plugging was 14.29 Pa. The elastic modulus of the TRG at the injection node after plugging was 19.42 Pa. The experimental results showed that the TRG with an elastic modulus of 19.42 Pa effectively plugged the fractures and remained stable for over 90 days under a pressure differential of 53 KPa, resulting in a 58.7% improvement in oil recovery compared with water flooding. However, it was difficult for the TRG with an elastic modulus of 14.29 Pa to plug fractures efficiently, and it only enhanced the oil recovery by 15.2%. The primary aim of this work was to establish a quantitative and normalized evaluation method for temperature-resistant gels (TRGs) used in fractured reservoirs. By introducing a classification system based on the elastic modulus (G′) and correlating it with the fracture scale and plugging performance, this study bridges the gap between laboratory gel evaluations and field applications. The results provide practical design criteria and contribute to improving the efficiency and adaptability of gel plugging strategies under harsh reservoir conditions.

## 1. Introduction

Carbonate reservoirs, formed through a combination of tectonic activity and ancient karst processes, account for over 60% of global oil reserves [1]. Carbonate reservoirs are primarily composed of calcium carbonate (CaCO_3_), with the occasional presence of dolomite (CaMg (CO_3_)_2_) [2,3]. This mineral composition influences the wettability, porosity, and potential chemical interactions with plugging agents. These reservoirs are characterized by low matrix permeability (typically less than 0.1 mD), and the main spaces for oil storage and flow are fractures and karst caves, which exhibit multiscale geometries and high permeability [4,5]. Due to the strong heterogeneity of the fractures, especially in the later stages of water flooding, the residual oil is primarily retained in fractures narrower than 1 mm [6,7].

The advancement of edge water and coning of bottom water often led to severe water channeling, resulting in early water breakthrough, increased water cut, and a significant decline in oil recovery efficiency [8,9]. The traditional water flooding methods are ineffective in recovering oil trapped in fine fractures, especially under complex reservoir conditions such as strong heterogeneity, high temperatures, high salinity, and great burial depths [10,11]. It is difficult to meet the challenges of plugging the dominant water channeling route in large-scale fractures and producing residual oil in fractures with a diameter of 1 mm or even less [12,13]. At present, a series of measures have been taken to solve the serious water channeling caused by bottom water coning in fractured reservoirs. Su et al. [14,15] studied the plugging behavior of foam in fractures. Subsequently, foam acid was used to clean up the blockage and inhibit the bottom water from coning, thereby establishing an effective oil and gas seepage channel. Chen et al. [16] used a combination of emulsion and nitrogen foam to plug the high seepage channeling and bottom water. Dordzieet et al. [17] used a nanoparticle-modified surfactant to form microemulsions with crude oil for improved oil recovery. Wang et al. [18] used a combination of gel and nano-microspheres to regulate and control complex fractures and caves in a hierarchical way. However, there are some problems in such reservoirs, such as strong heterogeneity, high temperatures, high salinity, and great reservoir depths. Using the above methods, it is difficult to plug the channels for a long time, and these methods have poor adaptability to environments with strong heterogeneity and poor oil–water selectivity [19].

Gel water plugging technology has emerged as one of the most effective and widely applied chemical methods in oil fields due to its high strength, good injectability, and cost efficiency [20]. Gels are formed by injecting polymer and crosslinking agent solutions into the reservoir, which subsequently crosslink to form a three-dimensional network structure that blocks high-permeability channels [21,22]. This technology allows the selective plugging of water pathways while maintaining oil flow channels [23,24,25,26]. The strength and performance of the gels can be tailored by adjusting the formulation, including the polymer concentration and crosslinker type [27,28]. Li et al. [29] developed a hydrophobic association polymer as a high-strength water plugging material to effectively plug fractures. Rubber particles are commonly used as flow regulators in fractured reservoirs. The inorganic particles have good chemical stability, high temperature resistance, and good mechanical strength [30]. Inorganic particles can withstand the harsh environments of fractured carbonate reservoirs. In addition, inorganic particles can control the fracture depth by controlling the particle size [31]. As a rigid material, inorganic particles can effectively plug water channeling channels, expand the sweep, and improve the oil recovery, and are good profile control agents [32]. Huang et al. [33] synthesized a functional FPL with multi-functional groups via micelle polymerization to plug micropores of different sizes. However, the injectability of rubber particles has always been a difficult problem in the application of fractured reservoirs. The gel system has become one of the most widely used and effective chemical water plugging methods in oil fields because of its low cost, high strength, and easy injection [34,35]. Therefore, a more effective method is still needed to evaluate the application rules of the gel in fracture plugging, so that the bottom water plugging process is carried out economically and efficiently.

In order to solve the problems of rapid increases in water content and sharp decreases in oil recovery caused by bottom water coning in fractured reservoirs, a multi-scale fracture model was established in the experiment, and the gelation performances of TRGs with different elastic moduli were evaluated. The strength of the TRGs was normalized using the G′/G″ method, and then the influence chart of different TRG strengths and different fracture scales on the bottom water breakthrough pressure gradient and plugging rate was established. The optimal function was obtained using MATLAB nonlinear surface fitting. Finally, the application experiment of the characteristic patterns chart was carried out. The experiments showed that after calculating the required plugging rate according to the core physical parameters, the gel plugging agent with appropriate strength can be selected in the chart. Specific plugging agents could be selected as required to effectively plug the bottom water energy of a fracture of a certain scale. The goal of plugging the dominant seepage channel was achieved.

## 2. Results and Discussion

### 2.1. Evaluation of Gelation Performance

**Figure 1:**
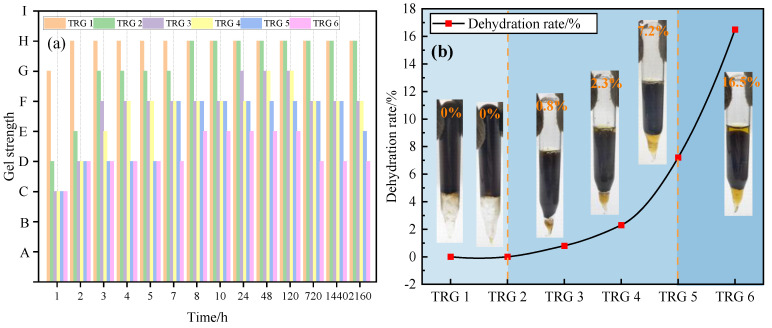
Evaluation of the gelation performance of the TRG at 130 °C for 90 d: (**a**) evaluation of the gelation time and gelation strength of the TRG; (**b**) evaluation of the aging stability of the TRG.

Figure 1 shows the gelation performance evaluation of the TRG at 130 °C over a 90 day aging period, simulating long-term reservoir conditions. As shown in Figure 1a, the gelation time of the TRG gradually increased while the gel strength decreased from TRG 1 to TRG 6. TRGs 1–5 completed gelation within 7 h, reaching strength levels of F to G, while TRGs 1–2 retained high strength (level H) even after 90 days of aging. This demonstrates excellent long-term mechanical stability under high-temperature conditions. In contrast, TRG 6 gelled more slowly (within 8 h) and reached only strength level E due to its lower polymer and crosslinker concentrations. Insufficient molecular interactions led to the formation of a loose colloidal dispersion gel (CDG), which was inherently weak and lacked a stable crosslinked network structure [36]. As the concentrations of the polymer and crosslinker decreased, the aging stability of the TRG correspondingly declined.

Figure 1b further illustrates this trend in terms of the dehydration rate. After 90 days of aging, TRGs 1–5 exhibited dehydration rates of between 0% and 7.2%, indicating excellent water retention and structural integrity. In contrast, TRG 6 had a significantly higher dehydration rate of 16.5%, reflecting poor long-term stability. This behavior can be attributed to the lower network density and less robust spatial structure of TRG 6, making it resistant to thermal degradation and water loss over time. These results suggest that TRGs with a higher initial elastic modulus and denser gel networks are more suitable for long-term applications in high-temperature fractured reservoirs.

### 2.2. Normalized Evaluation of TRG’s Elastic Modulus

**Figure 2:**
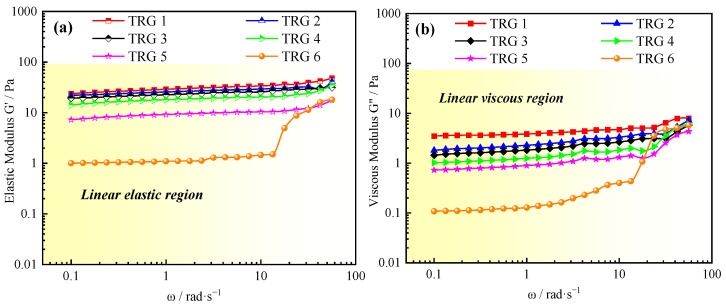
Dynamic viscoelastic curves of different elastic moduli of TRGs after gelation: (**a**) elastic modulus (G′); (**b**) viscosity modulus (G″).

The strength of a gel reflects its mechanical strength and water control ability, which are key indicators for oil field applications [37]. Rheological studies provide two important parameters, the elastic modulus (G′) and viscous modulus (G″). The elastic modulus (G′) represents the energy stored through the elastic deformation, while the viscous modulus (G″) represents the energy lost through the viscous deformation [38]. The elastic modulus of the TRG was normalized through the measurement results of rheological G′ and G″, as shown in Figure 2a,b. The experimental results showed that the G′ of the TRG was higher than the G″. This indicated that the elastic behavior of the TRG was dominant. As the concentrations of polymer and crosslinker increased, the G′ values rose significantly, indicating a more developed spatial network structure and stronger viscoelastic properties. For example, TRG 1 (highest concentration) reached a G′ value of 31.24 Pa, while TRG 6 (lowest concentration) exhibited only 1.45 Pa. This suggests that the material’s rigidity and resistance to deformation under stress were greatly enhanced at higher concentrations. The rigidity of the TRG material was enhanced. Furthermore, the weighted average method (which refers to the average value during steady-state shear) was used for the normalization of the G′ values at different TRG elastic moduli during the steady-state shear process. In conventional reservoirs, gels with an elastic modulus (G′) greater than 10 Pa are generally considered high-strength gels. However, the focus of this study was on fractured reservoirs. Therefore, the different strengths of TRG were divided according to the normalized elastic modulus (high-strength gel with a strength greater than 25 Pa, medium-strength gel with a strength between 10 and 25 Pa, and low-strength gel with a strength lower than 10 Pa) [39], as shown in Table 1. This classification provides a practical framework for evaluating and selecting TRGs for fractured reservoirs.

### 2.3. Patterns of Bottom Water Bearing Pressure

**Figure 3:**
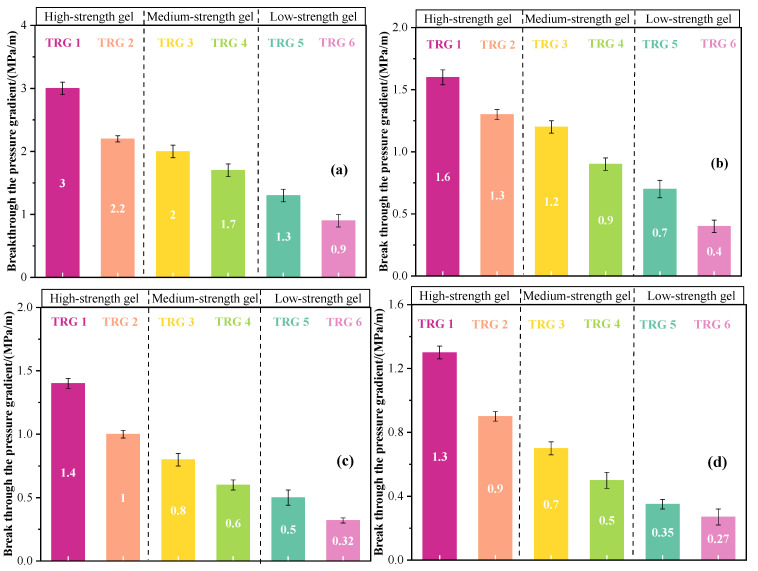
Characteristic curves of bottom water pressure-bearing patterns of TRGs: (**a**) 1 mm; (**b**) 5 mm; (**c**) 8 mm; (**d**) 10 mm.

The bottom water breakthrough pressure gradients of TRGs with varying elastic moduli were experimentally evaluated under multi-scale fracture conditions, as illustrated in Figure 3a–d. The results revealed a clear positive correlation between a TRG’s elastic modulus and breakthrough pressure. Specifically, stronger gels exhibited higher resistance to bottom water intrusion, indicating enhanced sealing performance. Under identical TRG strength, the breakthrough pressure gradient showed an inverse relationship with the fracture width. For fractures ranging from 1 mm to 10 mm, the measured breakthrough pressure gradients were as follows: 1.3–3 MPa/m (TRG 1), 0.9–2.2 MPa/m (TRG 2), 0.7–2.0 MPa/m (TRG 3), 0.5–1.7 MPa/m (TRG 4), 0.35–1.3 MPa/m (TRG 5), and 0.27–0.9 MPa/m (TRG 6). This behavior can be attributed to the viscoelastic nature of the gels. TRGs with higher G′ values possess denser three-dimensional networks, which contribute to higher rigidity and deformation resistance. However, due to their dominant elastic characteristics, TRGs tend to exhibit limited adhesion strength at the gel–rock interface. As the bottom water energy increases, shear forces may lead to the detachment of the gel from the fracture walls, enabling water to bypass the plugging agent via the interface [40].

### 2.4. Evaluation of Scouring Resistance

**Figure 4:**
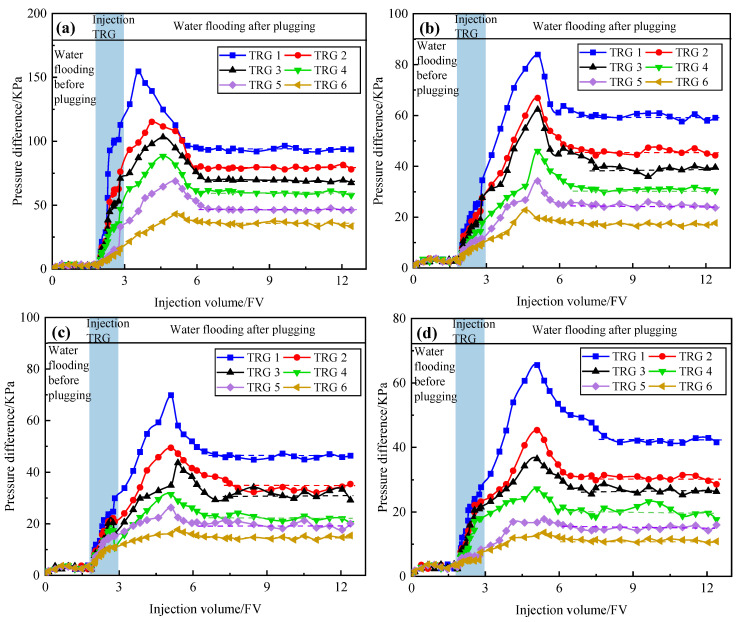
Characteristic curves of the scouring resistance levels of TRGs: (**a**) 1 mm; (**b**) 5 mm; (**c**) 8 mm; (**d**) 10 mm.

The plugging rates of TRGs with different elastic moduli under multi-scale fractures were measured to evaluate the scouring resistance, as shown in Figure 4a–d. Water flooding was performed until the pressure stabilized before TRG injection. Then, 1 PV of TRG was injected, followed by a second water flooding phase until the pressure again reached stability. The pressure initially increased to a peak and then dropped rapidly as the water injection continued. This trend reflected the breakthrough behavior and resistance of the gel. The results showed that the plugging rates of the TRGs increased with the gel strength. For the same TRG strength, the plugging rate decreased as the fracture width increased. Specifically, in 1–10 mm fractures, the plugging rates of TRGs 1–6 ranged from 92.96% to 96.79%, 90.17% to 96.24%, 88.95% to 95.71%, 85.09% to 94.97%, 79.76% to 93.54%, and 72.69% to 91.63%, respectively. In fractured reservoirs, a plugging rate above 90% is generally sufficient to meet the plugging requirements [4]. Based on the plugging performance patterns of TRGs 1–6, TRGs 1–2 are suitable for fractures smaller than 10 mm, TRG 3 is suitable for fractures smaller than 8 mm, TRG 4 for fractures smaller than 5 mm, and TRGs 5–6 are only applicable to fractures equal to or smaller than 1 mm. The pressure fluctuations observed during water injection were attributed to indirect secondary plugging caused by TRG migration and redeposition within the fracture network.

### 2.5. Establishment and Fitting of Characteristic Chart

**Figure 5:**
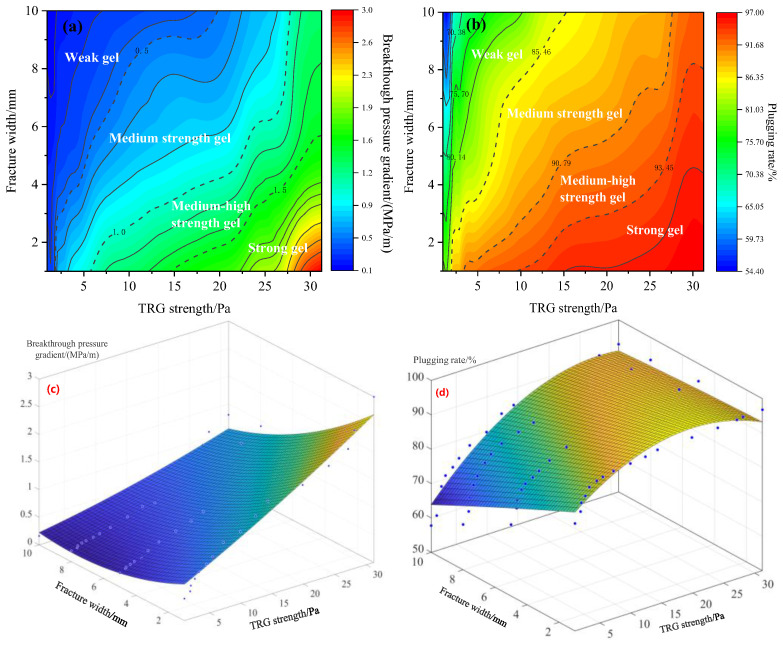
(**a**) The characteristic chart of bottom water breakthrough pressure gradients. (**b**) A chart of the plugging rates. (**c**) The three-dimensional fitting of the bottom water breakthrough pressure gradient characteristic chart. (**d**) The three-dimensional fitting of the plugging rate characteristic chart.

The related influence pattern chart was established experimentally to clarify the influence patterns of TRGs with different elastic moduli, as shown in Figure 5a,b. The related influence pattern chart was fitted with a three-dimensional surface using MATLAB R2022b (The MathWorks, Inc., Natick, MA, USA), as shown in Figure 5c,d. The optimal function fitting formula was obtained, as shown in Equation (1). The fitting formula for the optimization function is shown in Table 2. The TRGs were divided into four grades according to their strength, bottom water breakthrough pressure gradient, and plugging rate: strong gel (G′ ≥ 25 Pa, bottom water breakthrough pressure gradient ≥ 1.5 MPa/m, plugging rate ≥ 93%), medium–high-strength gel (G′ 10~25 Pa, bottom water breakthrough pressure gradient 1.0~1.5 MPa/m, plugging rate 90~93%), medium-strength gel (G′ 3~10 Pa, bottom water breakthrough pressure gradient 0.5~1.0 MPa/m, plugging rate 85~90%), weak gel (G′ < 3 Pa, bottom water breakthrough pressure gradient < 0.5 MPa/m, plugging rate < 85%). Fractures of different scales could be effectively plugged by TRGs with different elastic moduli. The plugging rate is more sensitive to changes in elastic modulus in smaller fractures (≤3 mm), where a 50% increase in G′ can improve the plugging rate by up to 4–6%. In contrast, for larger fractures (≥8 mm), the plugging rate is primarily influenced by the fracture width, and the same 50% increase in G′ results in less than 2–3% improvement. Interaction effects between the fracture scale and gel strength were also observed, confirming the need for accurate gel selection for specific fracture conditions. Therefore, the TRG with the appropriate elastic modulus was selected to plug the dominant seepage fractures and exploit the residual oil in the small fractures.

(1) $$z(x,y)=p_{00}+p_{10}\cdot x+p_{01}\cdot y+p_{20}\cdot x^{2}+p_{11}\cdot x\cdot y+p_{02}\cdot y^{2}$$

The left-hand side of the equation represents the breakthrough of the pressure gradient (MPa/m) (Figure 5c) and plugging (%)(Figure 5d); here, x represents the TRG strength (Pa) and y represents the fracture width (mm) in Figure 5c,d.

### 2.6. Application Experiment for Characteristic Charts

**Figure 6:**
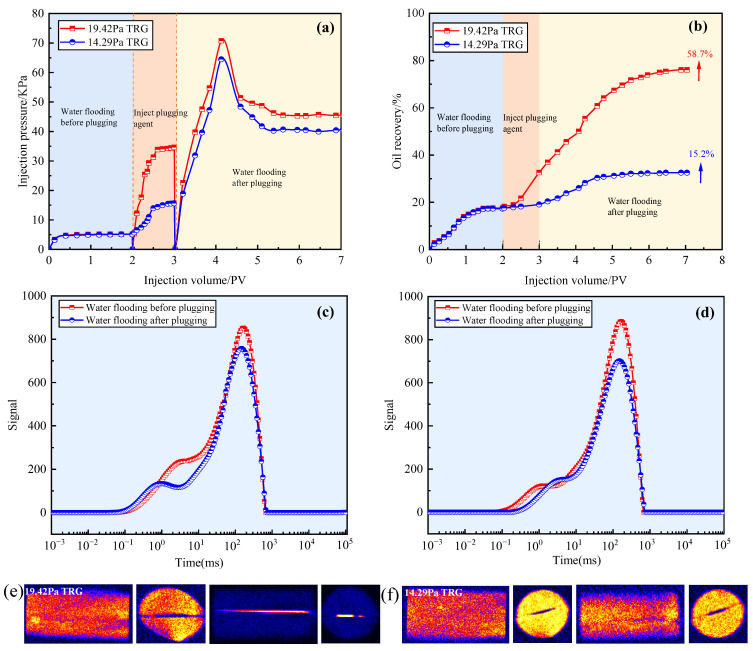
Effects of different plugging agents on plugging performance and oil recovery of fractured cores: (**a**,**b**) characteristic curves of pressure and oil recovery after TRG plugging with different elastic moduli; (**c**,**d**) effects of 19.42 Pa and 14.29 Pa TRGs on the T_2_ spectrum of fractured cores before and after plugging; (**e**,**f**) nuclear magnetic resonance images depicting the core states before and after plugging with 19.42 Pa and 14.29 TRGs.

Figure 6a,b shows the evaluation effects of the 19.42 Pa and 14.29 Pa TRGs on the plugging performance and oil recovery of fractured cores. The analysis in Figure 6a indicates that similar injection pressure characteristic curves were obtained after plugging with the 19.42 Pa and 14.29 Pa TRGs. However, the maximum pressure and stable pressure after injecting the 19.42 Pa TRG were higher than those obtained after injecting the 14.29 Pa TRG. The analysis in Figure 6b indicates that the oil recovery increased by 58.7% after plugging with the 19.42 Pa TRG. After plugging with the 14.29 Pa TRG, the oil recovery increased by 15.2%. This is because 19.42 Pa TRG can effectively plug the fractures in the core, so that the injected water can displace the remaining oil in the core matrix, thereby greatly improving the oil recovery. Due to its weak strength, the 14.29 Pa TRG cannot effectively plug the fractures in the core. The injected water gradually breaks through the TRG along the fracture and reaches a stable state. This is also the reason why the maximum pressure and stable pressure after injecting the 19.42 Pa TRG were higher than those after injecting the 14.29 Pa TRG.

Figure 6c,d show the T_2_ spectrogram curves of water flooding before plugging and water flooding after plugging. In each group, the signal of the macropore area decreased. This is due to the retention of plugging agent in the fractures after injection. After injecting 19.42 Pa TRG, the small aperture signal was greatly reduced. This is because the TRG effectively plugs the fractures in the core and enhances the sweep range of the injected fluid. However, after injecting 14.29 Pa TRG, the signal of the small aperture was not obviously enhanced, caused by the plugging agent not completely plugging the fracture. Figure 6e,f show the effects of the 19.42 Pa and 14.29 Pa TRGs on nuclear magnetic resonance images of fractured cores before and after plugging. The colors in the image correspond to the following different fluid phases: blue indicates weak hydrogen signals, while red indicates strong hydrogen signals. The water phase uses deuterium water, which has no hydrogen signals and appears blue. Crude oil and gel exhibit stronger hydrogen signals, appearing yellow or even red. The gel is primarily distributed within the fractures and appears yellow or red. These colors are used to visually distinguish the distribution of each fluid phase within the core. The image on the left shows the water flooding before plugging. The reason no water phase is displayed is that the injected water quickly flows through the fractures, while the oil phase remains largely present in the core, with the oil signal covering the water signal. The image on the right shows the water flooding after plugging, where a significant gel signal can clearly be observed within the fractures. This gel has blocked the water channel, allowing the subsequent water flooding to displace the crude oil from the core, meaning no signal can be detected in the core matrix. This indicates that the TRG with an elastic modulus of 19.42 Pa, which is above the plugging threshold for 1 mm fractures, can achieve effective plugging. In contrast, the TRG with an elastic modulus of 14.29 Pa still shows a large crude oil signal after plugging, which does not meet the plugging requirements. The experimental results are the same as those of the T_2_ spectrum.

## 3. Conclusions

In this study, a normalization method based on the elastic modulus (G′) was proposed to classify the strengths of temperature-resistant gels (TRGs), providing a more objective way to characterize gel performance beyond the conventional strength grading system (A-I) by linking it to plugging effectiveness. A fracture scale–gel strength correlation chart was developed. Using nonlinear surface fitting (MATLAB), an empirical formula based on this chart was constructed to guide the selection of gels for specific fracture widths and desired plugging rates, offering strong applicability for strategic implementation in oil field operations. A direct experimental validation of the gel strength thresholds was also conducted. It was demonstrated that TRGs with an elastic modulus greater than 19.42 Pa could effectively plug 1 mm fractures, whereas lower-strength gels (e.g., 14.29 Pa) failed, supporting a practical, G′-based design criterion. Through laboratory performance evaluations, the study established the direct applicability of the gelation time, gel strength, aging stability, and matching strategy to fractured reservoirs under harsh reservoir conditions (130 °C and 200,000 mg/L salinity). Under such reservoir conditions, the stable gelation time of the TRG is within 8 h, and the dehydration rate is less than 16.5% after 90 days, which indicates that the aging stability is strong. This research bridges the gap between gel development and field application, offering a reproducible and visual method for the selection and evaluation of TRGs in complex fractured reservoirs.

## 4. Materials and Methods

### 4.1. Materials

The temperature-resistant gel (TRG) used in this study was provided by Northwest Branch of China Petrochemical Company (Urumqi, China). The experimental temperature of 130 °C was selected to replicate the actual reservoir conditions of Sinopec Northwest Branch of China Petrochemical Company. It was used to include the polymer LH301 (chemical composition: polyacrylamide; hydrolysis degree of approximately 25–30%; molecular weight: 95 × 10^5^ Dal); the cross-linking agents were hexamethylenetetramine (HMTA), catechol (CL), and resorcinol. The NaCl (purity ≥ 99.5%), CaCl_2_ (purity ≥ 96.0%), and MgCl_2_ (purity ≥ 98.0%) were purchased from China Shanghai Aladdin Reagent Co., Ltd. The deionized water was prepared using a laboratory ultrapure water machine (Shanghai Hetai instrument co., ltd, ULUPURE, UPT-II, Shanghai, China). The total salinity of the formation water was 20 × 10^4^ mg/L (Table 3 shows the salt ion concentration). The carbonate rock cores were provided by Beijing Tiandi Kaiyuan Geological Technology Co., Ltd. (Beijing, China), and multi-scale fractures were made using table-type cutting machines (DEWALT, Stanley Black & Decker, Towson, MD, America). All reagents were used as received without further purification.

### 4.2. Preparation of the TRG

Firstly, a certain amount of LH301 was accurately weighed and slowly added to the simulated formation water, which was constantly stirred for 2 h to fully dissolve and swell. Subsequently, a certain amount of cross-linking agent was added and fully dissolved during the stirring process to prepare the original TRG solution. The TRG was aged in an oven at 130 °C after being placed in a bottle. The gelation performances of TRGs with different elastic moduli were recorded. The combined dosage of TRG was designed to prepare bulk gels with different elastic moduli, as shown in Table 4.

### 4.3. Determination of Gelation Performance

**Figure 7:**
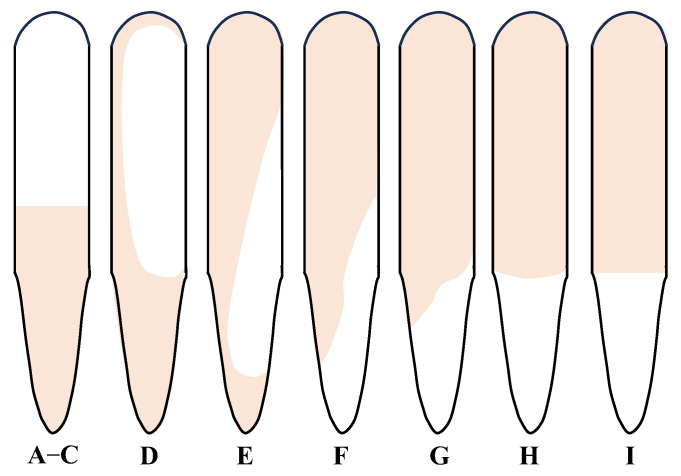
Schematic diagram of gel strength standard.

First, 21 mL of the original TRG solution prepared in Section 4.2 was put into a bottle, sealed, and put into an oven at 130 °C. The Sydansk bottle test method was used to determine the gelation time and gel strength of the TRG [41]. A standard schematic diagram of the gel strength is shown in Figure 7. The strength of the TRG was measured every 1 h. When the F strength was reached, this was the gelation time of the TRG [42]. The TRG was placed in an oven at 130 °C for at least 90 d to observe its thermal stability. The dehydration rate was the ratio of the precipitated liquid to the original TRG solution over a certain period of time [43]. According to the Equation (2):

(2) $$M=\frac{M_{1}-M_{2}}{M_{1}}\times 100\%$$

*M* is the dehydration rate, *M*_1_ is the original TRG solution mass, and *M*_2_ is the TRG mass after dehydration.

### 4.4. Rheology Measurement

In the experiment, a Haake Mars 60 (Thermo Fisher Scientific, Karlsruhe, Germany) rheometer was used to study the rheological properties of the TRG that reached the gelation time. The experiments were carried out using a common bottom plate and a flat rotor. The TRG was spread all over the bottom plate without overflowing (the normal force was maintained at 0.5 ± 0.1 N; a parallel plate geometry with a diameter of 40 mm was used, and the gap between the plates was set to 1 mm.) The G′/G″ of the TRG was measured over a frequency range of 0.1–100 rad/s. A normalized evaluation method for TRG strength was established. The TRGs with different elastic moduli were unified and quantified using the G′/G″, and then the TRG based on the normalized strength was divided.

### 4.5. Bottom Water Energy

**Figure 8:**
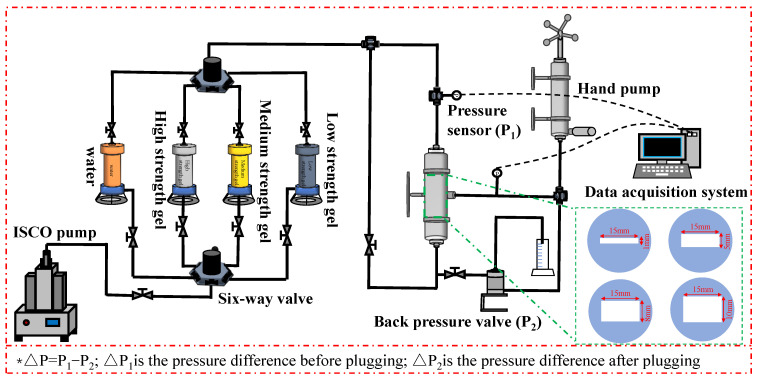
Experimental procedure chart of the bottom water bearing pressure levels of different plugging agents.

The experiment shown in Figure 8 was used to evaluate the bottom water pressure patterns of the TRGs with different elastic moduli. The core was placed vertically in the experiment. Before the experiment, N_2_ was used to evacuate the pipeline to prevent the liquid in the pipeline from interfering. The original TRG solution was injected into multi-scale fractures from above. The original TRG solution was prepared as outlined in Section 2.2. The fracture scale values were 1, 5, 8, and 10 mm. The injection amount of the TRG was 1 FV, and it was aged to its gelation time. The experimental temperature was set at 130 °C. The confining pressure was 2 MPa, and it was kept 2 MPa higher than the injection pressure. The back pressure was 0.5 MPa. The constant pressure gradient was used to simulate the bottom water energy [44], and the pressure increase speed was 0.01~0.05 MPa/min. The pressure range of the bottom water of the TRG was determined after aging at 130 °C until the gelation time. The bottom water breakthrough pressure is the pressure difference when the first drop of liquid flows out of the outlet ends [45]. The bottom water breakthrough pressure gradient at vertical depth is calculated according to the bottom water breakthrough pressure. The breakthrough pressure gradient of the bottom water is the ratio of the breakthrough pressure to the vertical depth of the core [46,47]. According to the Equation (3):

(3) $$G=\frac{\Delta P}{L}$$

*G* is the bottom water breakthrough pressure gradient, Δ*P* is the bottom water breakthrough pressure, and *L* is the vertical depth of the core.

### 4.6. Scouring Resistance of Gel

The original TRG solution was injected into multi-scale vertical fractures from above at a displacement of 1 mL/min. The selected fracture scale values were 1, 5, 8, and 10 mm. The injection amount of the TRG was 1 FV, and it was aged to its gelation time. The experimental temperature was set at 130 °C. The confining pressure was 2 MPa, and it was kept 2 MPa higher than the injection pressure. The back pressure was 0.5 MPa. The plugging rate is calculated according to the change of core permeability before and after TRG injection, which reflects the effectiveness of the gel in reducing fracture permeability [48]. It can be calculated according to Equation (4):

(4) $$Dw=\frac{\frac{1}{\Delta P_{1}}-\frac{1}{\Delta P_{2}}}{\frac{1}{\Delta P_{1}}}\times 100\%$$

Here, *Dw* is the plugging rate, Δ*P*_1_ is the pressure difference before plugging, and Δ*P*_2_ is the pressure difference after plugging.

### 4.7. Establishment of Characteristic Chart

According to the experimental study, the chart outlining the influences of different fracture scales and TRG elastic moduli on the pressure gradient of the bottom water and plugging rate was established. The goal of accurately guiding the selection and dosage of plugging agents under different application conditions was realized.

### 4.8. Verification of of Characteristic Chart

In order to verify the chart of characteristic patterns, displacement experiments of the TRGs with different elastic moduli on four core samples were carried out. The physical parameters of the core are shown in Table 5. The effect of plugging strength nodes on improving the oil recovery of the core matrix under high temperatures and high salt conditions was evaluated. According to the fracture permeability and matrix permeability of the core, theoretically the plugging rate of cores FM1 and FM2 were above 95.18% and 95.49%, which could improve the core matrix recovery. The specific experimental steps were as follows: (1) The core samples were dried at 65 °C for 24 h, and the initial oil saturation was established by vacuuming and pressurizing saturated crude oil; (2) A water flooding experiment was carried out, whereby simulated formation water (prepared from heavy water) was used to displace 4 cores. When the injection pressure in each stage was stable, the displacement solution was replaced. (3) The plugging experiment with the TRG elastic modulus node was carried out. FM1 was injected with a gel with a plugging rate of over 95.18% at 1 FV (elastic modulus of 19.42 Pa TRG). FM2 was injected with a gel with a plugging strength of lower than 95.49% at 1 FV (elastic modulus of 14.29 Pa TRG). The plugging agent was aged for 2 days after being injected to ensure that the TRG was fully formed. (4) A water flooding experiment was carried out (the water used was simulated formation water, prepared from heavy water). The tracking confining pressure was 2 MPa, the experimental temperature was 130 °C, and the injection rate was 0.5 mL/min. The injection pressure and oil displacement efficiency were recorded during the experiment. In the experiment, the initial oil saturation was established via vacuuming and pressurizing saturation. This was mainly to avoid the influence of conventional water flooding on the saturated oil effect by establishing the dominant channel formed by initial water saturation. The density and viscosity of crude oil are 0.93 g/mL and 14.5 mPa·s. The initial permeability of the fractured cores was obtained using the Poiseulle equation, as shown in Equations (5) and (6). Because the matrix permeability of each core was low, the matrix permeability was ignored and the fracture permeability was used.

(5) $$k_{t}=k_{m}+\sum k_{fi}\cos\alpha_{i}$$

Here, *k_t_* is the total permeability of the core, mD; *k_m_* is the permeability of the core matrix, mD; *k_fi_* is the permeability of the fracture *i* (permeability along the extension direction of the fracture), mD; α*_i_* is the included angle between the fracture *i* and the fluid seepage direction (°).

(6) $$k_{f}=\frac{\psi_{f}b^{2}}{12}$$

Here, *k_f_* is the fracture permeability, mD; ψ*_f_* is the fracture porosity, %; *b* is the fracture width, cm.

## Figures and Tables

**Table 1:** The normalized elastic moduli of the TRGs.

Normalized Gel	TRG	Gelation Time/h	Gelation Strength	Elastic Modulus (G′)
High-strength gel	TRG 1	1	G	31.24
	TRG 2	3	G	27.12
Medium-strength gel	TRG 3	3	F	24.03
	TRG 4	4	F	19.42
Low-strength gel	TRG 5	7	F	9.90
	TRG 6	8	E	1.45

**Table 2:** Parameter table of optimized function fitting formula.

Fitting Parameters	p_00_	p_10_	p_01_	p_20_	p_11_	p_02_	R^2^
c	0.7587	0.06169	−0.1632	0.0002879	−0.004365	0.01078	0.9670
d	85.45	1.430	−2.243	−0.03770	0.06810	−0.01139	0.9386

**Table 3:** Salt ion concentration of simulated formation water.

Na^+^	Ca^2+^	Mg^2+^	Cl^−^	Total Salinity/(mg/L)
71,243	10,011	10,003	111,271	202,528

**Table 4:** Combination table of dosages for different elastic moduli of TRGs.

Plugging Agent	NO.	Polymer LH301/%	HMTA/%	CL/%	Resorcinol/%
TRG	1	0.8	0.40	0.25	0.25
	2	0.7	0.35	0.20	0.20
	3	0.6	0.30	0.15	0.15
	4	0.5	0.25	0.10	0.10
	5	0.4	0.20	0.05	0.05
	6	0.3	0.15	0.075	0.075

**Table 5:** Physical parameters of the cores.

NO.	Fracture Scales/mm	Length/mm	Diameter/mm	Weight/g	Porosity/%	*k_f_*/mD	*k_m_*/mD	Initial Oil Saturation/%
FM1	1	50.21	25.01	48.40	20.80	6716	324	89.67
FM2	1	49.32	24.98	47.50	21.11	6716	303	90.23

## Data Availability

Data will be made available on request.
